# Inheritance of dicamba‐resistance in allotetraploid *Chenopodium album*

**DOI:** 10.1002/ps.7114

**Published:** 2022-08-17

**Authors:** Hossein Ghanizadeh, Kerry C Harrington, Lulu He, Trevor K James

**Affiliations:** ^1^ School of Agriculture and Environment Massey University Palmerston North New Zealand; ^2^ AgResearch, Ruakura Research Centre Hamilton New Zealand

**Keywords:** dicamba, genetic inheritance, herbicide resistance, polyploidy

## Abstract

**BACKGROUND:**

*Chenopodium album* L. is a troublesome weed in spring‐planted crops, and different levels of ploidy have been documented for this weed species. A population of *C. album* has evolved resistance to dicamba. The level of ploidy and inheritance of dicamba resistance was studied in this population.

**RESULTS:**

The resistant and susceptible individuals of *C. album* were confirmed as tetraploid by flow cytometry. Pair‐crosses were made between ten resistant and susceptible individuals. Eight F_1_ individuals from five crosses were confirmed resistant after treating with dicamba at 400 g a.e. ha^−1^. These individuals were selfed, and the response of their progenies to dicamba was assessed in dose–response experiments, and the results confirmed the resistance trait was dominant. Furthermore, an analysis of the segregation patterns revealed that the segregation response of all F_2_ progenies fitted a 3:1 (resistant/susceptible) ratio when treated with dicamba at 200, 400 and 800 g a.e. ha^−1^, suggesting a single gene was responsible for dicamba resistance.

**CONCLUSIONS:**

Dicamba resistance in the studied tetraploid population of *C. album* is governed by a single dominant gene. This type of inheritance suggests that selection for dicamba resistance can occur readily. © 2022 The Authors. *Pest Management Science* published by John Wiley & Sons Ltd on behalf of Society of Chemical Industry.

## Graphical abstract

Dicamba resistance in an allotetraploid *Chenopodium album* biotype is a dominant trait and governed by a single gene.

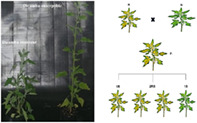

## 1 INTRODUCTION

Weeds are troublesome plant species when they grow alongside desirable crops. Weeds can compete with crops and result in significant yield losses if they are not controlled.^1^ The application of herbicides is the weed management strategy most commonly used by farmers and growers.^2^ However, the selection pressure exerted by herbicides on weed populations can be intense, resulting in an increased abundance of genes conferring resistance to herbicides in weed populations.^3^, ^4^ The evolution of resistance to herbicides in weeds will render the herbicides less effective. Given that only a limited number of chemical options are available for some crops and no new herbicidal mode of action has been commercialized in recent years, the evolution of herbicide resistance in weeds is concerning.^5^

Synthetic auxinic herbicides were the first herbicide group that was widely commercialized for weed management, with 2,4‐D (2,4‐dichlorophenoxyacetic acid) being the first of these synthetic herbicides introduced in 1945.^6^ Despite the prolonged application of synthetic auxinic herbicides, resistance to them has been documented in fewer weed species than acetyl‐CoA carboxylase (ACCase) and acetolactate synthase (ALS) inhibiting herbicide groups.^7^ It has been proposed that lower mutation rates, functional redundancy of auxin receptors in plants and fitness penalties associated with synthetic auxinic herbicide resistance have contributed to the low incidence of resistance recorded for these herbicides.^8^, ^9^ To date, only about 40 weed species have been reported as having evolved resistance to synthetic auxinic herbicides.^7^

*Chenopodium album* L., a member of the Amaranthaceae family, is an annual weed species that is ranked as one of the ten worst weeds in agricultural cropping systems.^10^ It is troublesome in spring‐planted crops such as maize (*Zea mays* L.) and sugar beet (*Beta vulgaris* L.).^11^, ^12^, ^13^ Dicamba is a synthesized auxinic herbicide that controls a wide range of broadleaved weed species selectively in several crops.^14^ In New Zealand, dicamba has been used to manage weed species such as *C. album* in maize and brassica crops.^12^, ^15^, ^16^ Dicamba‐resistant *C. album* was first reported from a maize field in New Zealand in 2005,^17^ and to the best of our knowledge, this is the only case of dicamba resistance recorded for this species globally.^18^ Previously, we showed that resistant *C. album* was almost ten times more resistant to dicamba compared to a susceptible population,^18^ and the mechanism of resistance was not associated with either reduced dicamba absorption/translocation or enhanced dicamba metabolism.^19^ In addition, it was noted that the resistant *C. album* suffers from a fitness cost.^20^

Inheritance studies are useful for discovering how herbicide resistance traits are being transmitted from one generation to another.^21^ Inheritance of herbicide resistance traits in weeds can be either cytoplasmic (i.e. genes governing the resistance traits are located in the cytoplasm) or nuclear (i.e. genes governing the inheritance traits are present inside the nucleus), with nuclear inheritance being the most common mode of inheritance among herbicide‐resistant weeds.^9^, ^21^ In addition, herbicide resistance can be inherited as dominant, semi‐dominant or recessive traits and can be governed by either single or multiple genes.^9^ Thus, the mode of inheritance can be varied and needs to be investigated for each case of herbicide resistance to improve our understanding of how resistance traits will be transferred to subsequent generations.^21^ Such information is necessary for designing appropriate strategies to minimize herbicide resistance gene migration and persistence in weed populations.

Polyploidy is a condition in which an organism possesses more than two sets of chromosomes in the nucleus.^22^ Polyploid plant species are primarily classified into autopolyploids (i.e. chromosome sets derived from a single parental species) and allopolyploids (i.e. the sets of chromosomes derived from different parental species).^23^ Polyploidy has been documented in several problematic weed species.^24^ In *C. album*, different levels of ploidy have been documented, namely diploid (2*n* = 2*x* = 18), tetraploid (2*n* = 4*x* = 36) and hexaploid (2*n* = 6*x* = 54).^25^ In addition, it has been noted that *C. album* is an allopolyploid species.^26^ To the best of our knowledge, there is only one case of herbicide‐resistant *C. album* for which the mode of inheritance was investigated; however, the authors did not report the ploidy level of the resistant *C. album*.^27^ In addition, the mode of inheritance in polyploid herbicide‐resistant weeds has rarely been investigated. In this research, we determined the level of ploidy and the mode of inheritance in dicamba‐resistant *C. album*.

## 2 MATERIALS AND METHODS

### 2.1 Plant material

The dicamba‐resistant (R) and ‐susceptible (S) populations used in this research originated from the Waikato region in New Zealand and have been described and characterized previously.^18^ Seeds of the R population used in this research were collected from plants that survived 1600 g a.e. ha^−1^ of dicamba.

### 2.2 Species identification using diagnostic chloroplast and ribosomal nuclear markers

**Figure 1:**
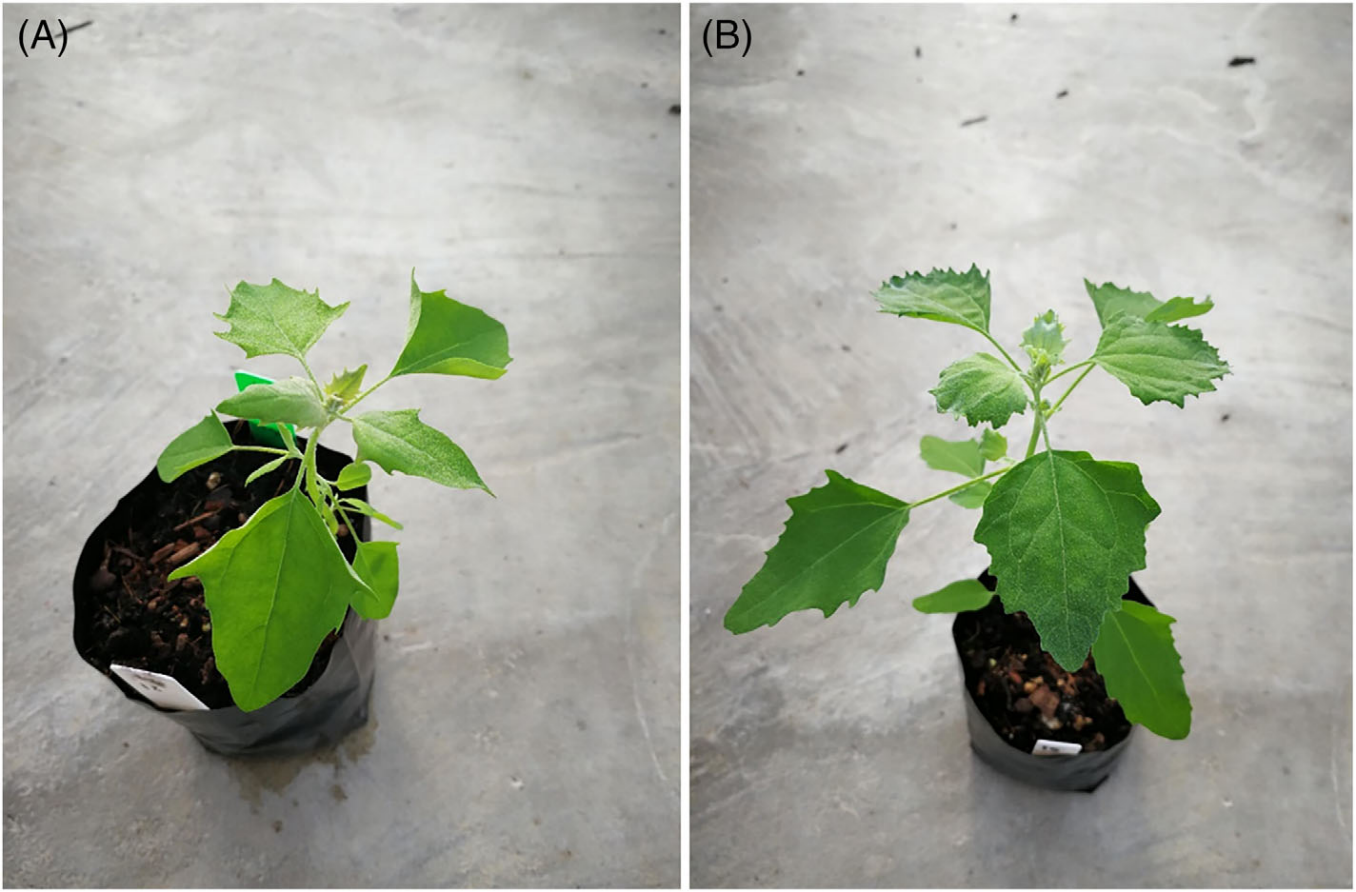
Variation in leaf morphology between (A) dicamba‐resistant and (B) dicamba‐susceptible *Chenopodium album*.

Previously, we reported that dicamba‐resistant *C. album* has some morphological features that differ from S plants (Fig. 1).^17^, ^20^ Given these differences in morphology, we checked if R and S phenotypes are still the same species and can interbreed to produce offspring after reciprocal pair‐crossing. For this, the chloroplast *psbA*‐*trnH* intergenic spacer region^28^ and internal transcribed spacers (ITS) that flank the 5.8 s nuclear ribosomal DNA region of plant genomes^29^ were sequenced and compared between R and S phenotypes. Fresh leaf tissues were collected from each phenotype, and DNA extraction was performed from these leaf tissues using the DNeasy Plant Mini kit (Qiagen, USA), following the manufacturer's instructions. DNA samples were then amplified using an iNtRON PCR kit (JH Science, Kirkland, WA, USA). To amplify the regions, previously published primers for the nuclear ribosomal DNA (*psbA*F: 5′‐GTTATGCATGAACGTAATGCTC‐3′ and *trnH*R: 5′‐CGCGCATGGTGGATTCACAAATC‐3′)^28^ and ITS (ITS5: 5′‐GGAAGTAAAAGTCGTAACAAGG‐3′ and ITS2: 5′‐TCCTCCGCTTATTGATATGC‐3′)^29^ regions were used. The polymerase chain reaction (PCR) total reaction volume was 15 μL, consisting of 0.2 μL of iTaq DNA polymerase, 2 μL of 10× PCR buffer, 1.5 μL of magnesium chloride (MgCl_2_), 2 μL of deoxyribonucleotide triphosphate (dNTP), 10 pmoles of the forward and reverse primers and 10–50 ng of DNA, and nuclease‐free water to bring the volume of reaction to 15 μL. The thermocycling protocol consisted of a period of initial denaturation at 94 °C for 4 min, 35 cycles of denaturation at 94 °C for 30 s, annealing at 55 °C for 30 s, extension at 72 °C for 45 s, and a 72 °C final extension period for 20 min. PCR products were then sequenced using the same primers for the chloroplast intergenic spacer and ribosomal nuclear regions. Subsequently, the sequence regions were compared against sequences from GenBank, administered by the National Center for Biotechnology Information (NCBI).

### 2.3 Ploidy analysis

The ploidy level of R and S plants were assessed using flow cytometry. For this, 50 plants of each population were grown from seeds. The seeds were pregerminated using the method described previously,^18^ and the seedlings were planted in 0.4‐L polyethene planter bags (diameter = 7 cm) containing potting media and slow‐release fertilizer as described by Ghanizadeh and Harrington.^12^ There was one plant in each planter bag and these were maintained in a glasshouse with average daily maximum/minimum temperatures of 20.1/17.8 °C and relative humidity (RH) of 53%. When plants were at the six‐ to seven‐leaf stage, leaf samples were collected from individual plants. Collected leaves were immediately wrapped in a wet paper towel and placed in a zip‐lock bag. The leaf samples (*n* = 50 for each population) were analysed using the method described by Otto.^30^ Briefly, a staining solution was prepared by mixing 2 mg mL^−1^ of 4,6‐diamidino‐2‐phenylindole (DAPI) with saturated dibasic sodium phosphate (Na_2_HPO_4_) to give a final concentration of DAPI of 2.5 μg mL^−1^. The leaf sample from each individual was chopped finely with a razor blade in 400 μL of extraction buffer (citric acid 0.5%; Tween 0.5%) and filtered through 30‐μm mesh filters (1‐ Partec, Christchurch, New Zealand). A 1.6 mL aliquot of the staining solution was then added, and the samples were left to stand for at least 2 min. All samples were co‐chopped with leaf tissue of *Trifolium repens* L. as an internal standard to provide an unbiased relative measure of DNA content. Only readings with a coefficient of variation (CV) of less than 3% were used to minimize experimental error. The samples were analysed using a CyFlow Space flow cytometer (2‐ Sysmex, Norderstedt, Germany). Data were analysed using Flowmax software. The ploidy level of the *C. album* samples was estimated using the average amount of DNA in a haploid genome (1C value) of diploid C. *album* ^25^ and the 1C value of *T. repens* (https://cvalues.science.kew.org).

### 2.4 Initial reciprocal pair‐crossing

Fifteen plants of each population were grown using the method described earlier. The plants were kept in a glasshouse with average daily maximum/minimum temperatures of 21.3/18.5 °C and RH of 55%. *Chenopodium album* is a predominantly self‐pollinating species, though up to 3% cross‐pollination has been recorded for this species.^31^ In our primary attempt, the flowers were emasculated under a microscope prior to reciprocal pair‐crosses between R and S plants. However, the emasculated flowers failed to produce seeds. Therefore, as an alternative approach, the flowers were pollinated manually. For this, pollen collected from the donor plants was transferred to the recipient ones using a small, sterile paintbrush. The pollinated flowers were bagged each time after pollen transformation. As the flowers of *C. album* are protogynous,^27^ the manual pollination of flowers allowed fertilization of stigma prior to anther dehiscence. The pair‐crosses were only made for apical flowers, and the flowers were immediately covered with pollen‐proof bags after pollination. The pair‐crosses were made between ten individuals of each population for 1 week. There were also five individuals of each population that were maintained in separate cages to obtain self‐pollinated seeds. At maturity, seeds were collected from all individuals and kept in separate bags until the next stage.

### 2.5 Phenotyping the pair‐crosses

The progenies of all ten pair‐crosses were grown using the method described earlier. Our primary investigation showed that the recommended field rate (400 g a.e. ha^−1^) of dicamba (Kamba 500, as dimethylamine salt) could be used as a discriminative dose to separate R from S individuals. The plants were kept in a glasshouse with average daily maximum/minimum temperatures of 23.5/19.7 °C and RH of 50%. When plants were at the four‐leaf stage, they were treated with 400 g a.e. ha^−1^ of dicamba using a laboratory track sprayer calibrated to deliver 230 L ha^−1^ of herbicide solution at a pressure of 200 kPa. The treated plants were evaluated 4 weeks after spraying. The average daily maximum/minimum temperatures in the glasshouse during the 4 weeks following treatment were 23.2/18.5 °C, and the RH was 53%. The individuals collected from R mother plants were all resistant to dicamba at the applied rate. However, eight individuals from five S mother plants appeared to be resistant to dicamba at the applied rate as they did not develop any symptoms typically observed for S plants (e.g. epinasty). Those eight individuals were kept in separate pollen‐proof cages and allowed to self‐pollinate to produce the second filial (F_2_) generation.

### 2.6 Dose–response analysis on the F_2_ generation

The response to dicamba of F_2_ families, R and S populations was assessed in dose–response experiments. For this, plants were grown from pregerminated seeds sown in 3‐L polyethylene planter bags (diameter = 18 cm) containing potting media and slow‐release fertilizer. There were 12 plants in each planter bag. Plants were maintained in a glasshouse with average daily maximum/minimum temperatures of 22.1/17.4 °C and RH of 55%. When the plants were at the four‐leaf stage, they were treated with dicamba rates of 0, 50, 100, 200, 400, 800, 1600, 3200, 6400 and 12 800 g a.e. ha^−1^. Dicamba was applied using a laboratory track sprayer calibrated to deliver 230 L ha^−1^ of herbicide solution at a pressure of 200 kPa. The average daily maximum/minimum temperatures in the glasshouse during the 4 weeks following treatment were 21.6/17.8 °C, and the RH was 58%. The response (mortality *versus* survival) of plants to dicamba treatments was evaluated at 4 weeks after treatment. This experiment was conducted in a randomized design with three replicates (i.e. three planter bags) and was repeated using the same aforementioned method.

### 2.7 Assessing the segregation patterns

To assess the segregation patterns of F_2_ families, plants were grown from pregerminated seeds in 3‐L polyethylene planter bags using the method outlined earlier. There were 10 to 12 plants in each bag and 6–8 bags for each herbicide rate (depending on the availability of seeds). Plants were maintained in a glasshouse with average daily maximum/minimum temperatures of 23.6/19.4 °C and RH of 50%. When the plants were at the four‐leaf stage, they were treated with dicamba rates of 0, 200, 400, and 800 g a.i. ha^−1^ using the method outlined earlier. The response of plants to dicamba was assessed 4 weeks after treatment. The average daily maximum/minimum temperatures in the glasshouse during the 4 weeks following treatment were 22.6/18.9 °C, and the RH was 52%. This experiment was conducted twice in time.

### 2.8 Statistical analyses

The data from dose–response experiments were checked for the assumptions of non‐linear regression prior to a two‐stage meta‐analysis approach using Sigmaplot (version 14.5). The data were fitted to a three‐parameter log‐logistic model:

$Y=\left[d/1+\exp\left(b\left(\log\left(x\right)-\log\left({\mathrm{LD}}_{50}\right)\right)\right)\right]$

where *Y* denotes plant survival as a percentage of the untreated control; *d* denotes the upper limit; *x* is herbicide rate; *b* denotes the slope around LD_50_ and LD_50_ denotes the herbicide rate corresponding to 50% reduction in plant survival.

The segregation of F_2_ families was tested against a single‐gene model using a two‐sided exact binomial test that used the method of small *P*‐values. The expected survival values corrected for the survival of the F_2_ progenies using the method outlined by Busi and Powles.^32^ In addition, a chi‐square test of homogeneity was conducted to determine if all F_2_ families had a similar segregation response to dicamba treatments.

## 3 RESULTS AND DISCUSSION

### 3.1 Species confirmation

To confirm the individuals of R population were correctly identified as *C. album*, the *psbA*‐*trnH* and ITS regions were sequenced as these regions have been used as barcodes in plant taxonomy to identify plant species.^28^, ^29^, ^33^ The results showed that the sequence from both R and S populations were identical to each other for the analysed *psbA*‐*trnH* and ITS regions, and also matched GenBank entries for *C. album* (e.g. JN044279.1 and for MH711165.1 *psbA*‐*trnH* and ITS regions, respectively). These results imply that the morphological features (i.e. abnormal leaf shape) manifested by the dicamba‐resistant *C. album* are likely to be associated with the mechanisms of resistance. Abnormalities in leaf shape and development have been documented in some other synthetic auxinic herbicide‐resistant weeds species such as *Soliva sessilis* Ruiz & Pav.^33^ and *Sinapis arvensis* L.^34^

### 3.2 The level of ploidy in dicamba‐resistant *C. album*

**Figure 2:**
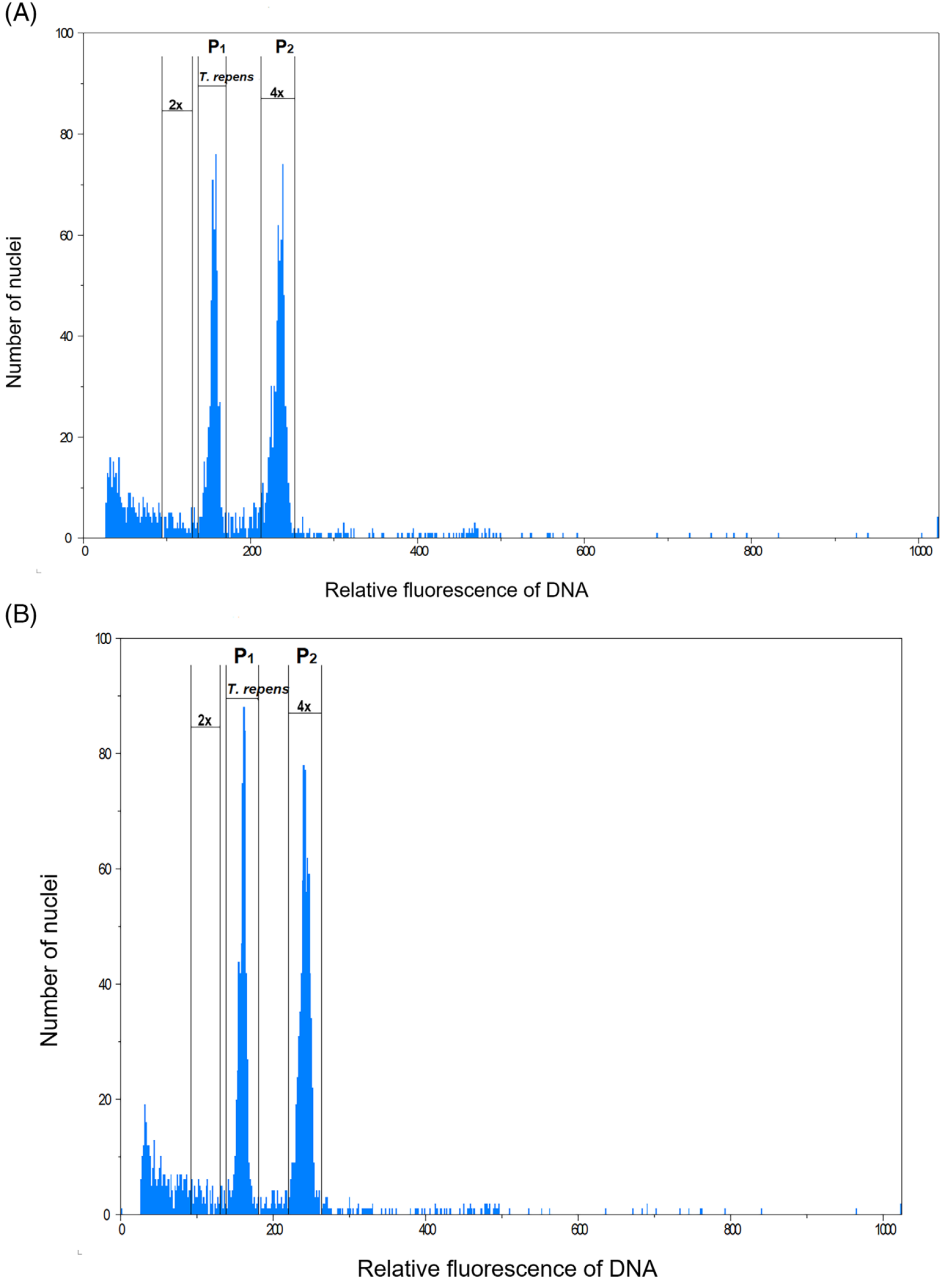
Flow cytometric histograms representing nuclear DNA content of nuclei released by fresh leaf tissue of (A) dicamba‐susceptible and (B) dicamba‐resistant *Chenopodium album*. *Trifolium repens* as an internal standard to provide an unbiased relative measure of DNA content. The peaks marked as P1 and P2 represent DAPI‐stained nuclei at G1‐phase and G2‐phase.

Flow cytometry was used to determine the ploidy of both R and S populations of *C. album* (Fig. 2). Analysis of the nuclei isolated from leaf samples of both R and S populations yielded a distinct peak at the relative fluorescence channel value of 233.0, which was almost 1.5 times greater than that of the *T. repens* (153.3), indicating that the genome size of both populations of *C. album* was 1.5 times bigger than that of the *T. repens. Trifolium repens* has a 1C value of 1.06^35^; thus, the average 1C value of samples from both R and S populations of *C. album* was estimated to be 1.59, which is twice the amount reported for diploid *C. album*.^25^ These results indicate that the evaluated samples from both populations were all tetraploids.

### 3.3 Dicamba resistance in *C. album* is dominant

**Figure 3:**
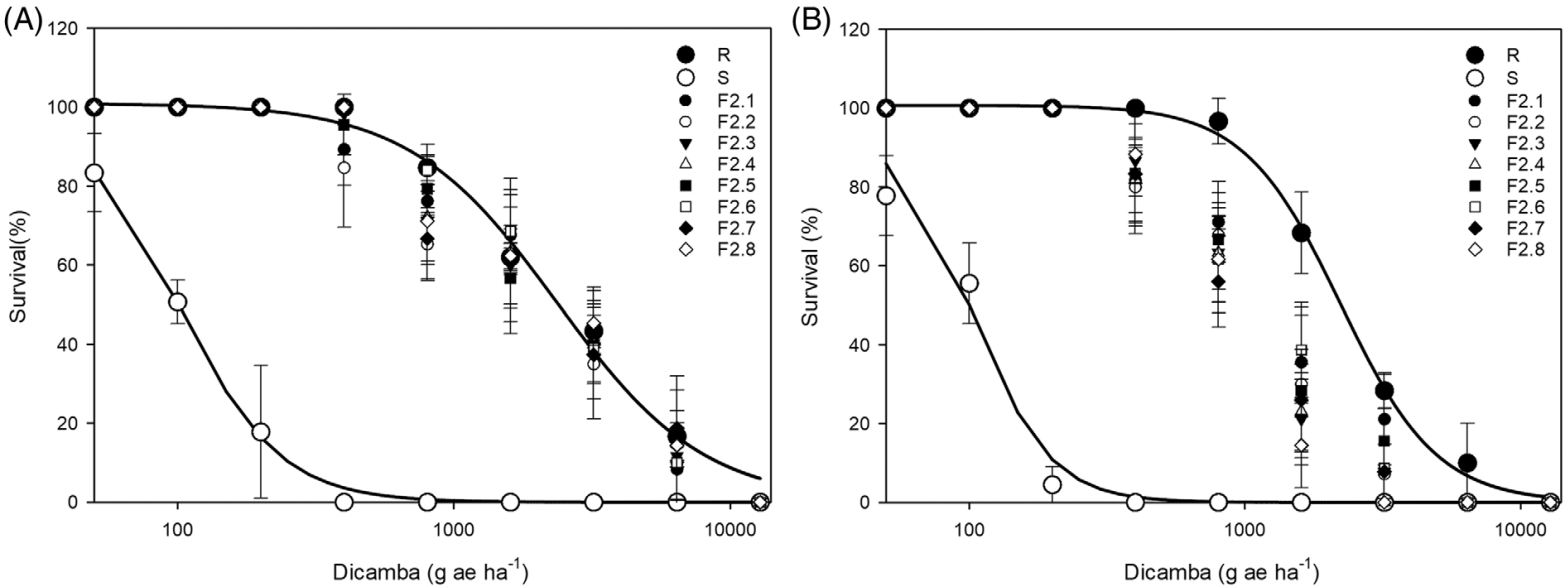
Dicamba dose–response of resistant (R), susceptible (S) and the progenies of eight F_2_ families (F2.1–F2.8) of *Chenopodium album* in the first (A) and second (B) experiments. The percentage survival of treated plants was used to produce the fitted curves. Vertical bars represent standard deviation of the mean.

All of the eight individuals collected from five F_1_ S mother plants manifested the phenotypic characteristics (e.g. less jagged leaves) observed for the R phenotype, suggesting that the morphological features associated with dicamba resistance in *C. album* are inheritable. Dose–response experiments were conducted to compare the response of F_2_ progenies with their R and S populations (Fig. 3(A,B)). At the recommended rate of dicamba (400 g a.e. ha^−1^) 100% mortality was recorded for the S population, while no mortality was recorded for the R population at this rate. At the recommended rate of dicamba, the percentage of survival was above 90 and 80% for all F_2_ progenies in the first and second dose–response experiments, respectively, and the response of all F_2_ families to dicamba was consistent, suggesting that the R parents were likely homozygous for dicamba resistance. In addition, the response of F_2_ progenies to all applied rates of dicamba was similar to that of the R phenotype, suggesting that dicamba resistance in *C. album* is a dominant trait with no evidence of maternal inheritance present.

Resistance to synthetic auxinic herbicides in most cases has been shown to be controlled by dominant resistance genes.^21^ For instance, resistance to dicamba and 2,4‐D in *Bassia scoparia* (L.) A. J. Scott^36^ and *Sisymbrium orientale* L.,^37^ respectively, was a dominant trait. However, to date, recessive inheritance has only been reported for picloram‐resistant *Centaurea solstitialis* L.^38^ and quinclorac‐resistant *Galium spurium* L.^39^ If a herbicide resistance trait is dominant, it will spread more rapidly compared to recessive ones as resistance traits can be manifested in both homozygous and heterozygous individuals.^21^, ^32^, ^40^, ^41^, ^42^ In all cases reported to date, resistance to auxinic herbicides was encoded in the nuclear genome, and it was not maternally inherited.^21^ Maternal inheritance has primarily been reported for target‐site based resistance to herbicides that inhibit photosystem II.^3^ Nuclear inheritance enables herbicide resistance alleles to be spread by pollen and seeds, though in predominantly autogamous species such as *C. album*, herbicide resistance alleles are primarily spread by seed.

### 3.4 Dicamba resistance in *C. album* is governed by a single gene

The segregation of F_2_ families was tested at three rates of dicamba, namely 200, 400 and 800 g a.e. ha^−1^, against a single‐gene model, with the assumption that the resistance gene presents in one of the homoeologous chromosomes (Table 1). The segregation pattern of all F_2_ families was similar in both experiments for the tested model. At all applied rates, the progenies from selfed F_1_ individuals were segregated, and the response of all families was not different according to a test of homogeneity, further indicating that the parents were all homozygous for dicamba resistance. The results also showed that the model fitted the response of all F_2_ families from all crosses at all dicamba rates, suggesting that dicamba resistance in *C. album* is controlled by a single gene. The *x* ^*2*^ value for heterogeneity of resistance segregation was not significant (Table 1), and the data from all F_2_ families were pooled within each rate. The G‐test of pooled data was not significantly different from a single‐gene model at all applied dicamba rates, suggesting that a single gene was contributing to dicamba resistance in *C. album*.

**Table 1:** Segregation for dicamba resistance at 200, 400 and 800 g a.e. ha^−1^ in second filial (F_2_) populations generated from crosses between resistant (R) and susceptible (S) *Chenopodium album* plants for a single‐gene model, with the assumption that the resistance gene presents in one of the homoeologous chromosomes (i.e*.* 3:1 segregation pattern)

	200 g a.e. ha^−1^					400 g a.e. ha^−1^					800 g a.e. ha^−1^						
Families	Treated	R	S	G	*P*	Treated	R	S	G	*P*	Treated	R	S	G		*P*	
*First experiment*										
R	50	50	0			50	50	0			50	42	8				
S	50	6	44			50	0	50			50	0	50				
F2.1	69	51	18		0.39	55	46	9		0.16	79	56	23			0.16	
F2.2	78	64	14		0.49	63	48	14		0.88	56	36	20			0.89	
F2.3	45	37	8		0.59	52	41	11		0.63	48	30	18			0.99	
F2.4	62	43	19		0.12	74	52	22		0.34	70	50	20			0.17	
F2.5	55	40	11		0.33	45	36	9		0.49	52	37	15			0.25	
F2.6	68	50	18		0.38	70	55	15		0.58	65	45	20			0.37	
F2.7	59	43	16		0.35	65	44	21		0.19	55	30	25			0.21	
F2.8	72	50	20		0.08	69	55	14		0.41	70	47	23			0.54	
Total	508	378	124		0.05	493	377	115		0.46	495	331	164			0.77	
Homogeneity (x^*2*^)				5.45	0.60				7.03	0.42				6.18		0.52	
*Second experiment*																		
R	50	50	0			50	50	0			50	40		10				
S	50	5	45			50	0	50			50	0		50				
F2.1	45	36	9		0.85	46	34	12		0.99	49	35		14			0.24	
F2.2	68	54	14		0.88	59	42	17		0.55	66	46		20			0.31	
F2.3	55	46	9		0.41	62	51	11		0.24	58	37		21			0.99	
F2.4	52	40	12		0.87	64	52	12		0.31	67	43		24			0.90	
F2.5	45	38	7		0.37	45	30	15		0.22	58	38		20			0.79	
F2.6	78	63	15		0.68	65	49	16		0.99	65	43		22			0.70	
F2.7	69	51	18		0.39	62	48	14		0.77	63	42		21			0.60	
F2.8	64	51	13		0.88	72	50	22		0.28	61	41		20			0.59	
Total	476	379	97		0.45	475	356	119		0.99	487	325		162			0.09	
Homogeneity (*x* ^*2*^)				2.88	0.89				6.60	0.47					1.22		0.99	

Note: R, resistant; S, susceptible; F2.1–F2.8, second filial generation number one to eight; G, maximum likelihood statistical significance G‐test; *P* , *P*‐value.

To further confirm that the resistance gene presents in only one of the homoeologous chromosomes, a second model that assumes a single gene that is present in both homoeologous chromosomes in an allotetraploid species (15:1 segregation ratio) was fitted to the F_2_ data (Table 2). The results showed that the second model did not fit the F_2_ data from all crosses at 200 and 400 g a.e. ha^−1^ of dicamba in both experiments. At 800 g a.e. ha^−1^ of dicamba, four and two F_2_ families fitted the expected response for a 15:1 segregation pattern in the first and second experiments, respectively. However, the second model failed to fit the pooled data from the F_2_ families treated with 800 g a.e. ha^−1^ of dicamba. These results imply that the resistance gene was present in one of the homoeologous chromosomes.

**Table 2:** Segregation for dicamba resistance at 200, 400 and 800 g a.e. ha^−1^ in second filial (F_2_) populations generated from crosses between resistant (R) and susceptible (S) *Chenopodium album* plants for a single‐gene model, with the assumption that the resistance gene presents in both homoeologous chromosomes (i.e*.* 15:1 segregation pattern)

	200 g a.e. ha^−1^					400 g a.e. ha^−1^					800 g a.e. ha^−1^				
Families	Treated	R	S	G	*P*	Treated	R	S	G	*P*	Treated	R	S	G	*P*
*First experiment*								
R	50	50	0			50	50	0			50	42	8		
S	50	6	44			50	0	50			50	0	50		
F2.1	69	51	18		<0.01	55	46	9		<0.01	79	56	23		0.09
F2.2	78	64	14		<0.01	63	48	14		<0.01	56	36	20		0.01
F2.3	45	37	8		<0.01	52	41	11		<0.01	48	30	18		0.01
F2.4	62	43	19		<0.01	74	52	22		<0.01	70	50	20		0.14
F2.5	55	40	11		<0.01	45	36	9		<0.01	52	37	15		0.17
F2.6	68	50	18		<0.01	70	55	15		<0.01	65	45	20		0.07
F2.7	59	43	16		<0.01	65	44	21		<0.01	55	30	25		<0.01
F2.8	72	50	20		<0.01	69	55	14		<0.01	70	47	23		0.02
Total	508	378	124		<0.01	493	377	115		<0.01	495	331	164		<0.01
Homogeneity (*x* ^*2*^)				5.45	0.60				7.03	0.42				6.18	0.52

*Second experiment*															
R	50	50	0			50	50	0			50	40	10		
S	50	5	45			50	0	50			50	0	50		
F2.1	45	36	9		<0.01	46	34	12		<0.01	49	35	14		0.2
F2.2	68	54	14		<0.01	59	42	17		<0.01	66	46	20		0.09
F2.3	55	46	9		<0.01	62	51	11		<0.01	58	37	21		<0.01
F2.4	52	40	12		<0.01	64	52	12		<0.01	67	43	24		<0.01
F2.5	45	38	7		0.01	45	30	15		<0.01	58	38	20		0.02
F2.6	78	63	15		<0.01	65	49	16		<0.01	65	43	22		0.02
F2.7	69	51	18		<0.01	62	48	14		<0.01	63	42	21		0.03
F2.8	64	51	13		<0.01	72	50	22		<0.01	61	41	20		0.04
Total	476	379	97		<0.01	475	356	119		<0.01	487	325	162		<0.01
Homogeneity (*x* ^*2*^)				2.88	0.89				6.60	0.47				1.22	0.99

Note: R, resistant; S, susceptible; F2.1–F2.8, second filial generation number one to eight; G, maximum likelihood statistical significance G‐test; *P* , *P*‐value.

Depending on the herbicide resistance mechanism, various patterns of segregation have been documented in weeds resistant to synthetic auxinic herbicides. In resistant weeds with a modification in the target site of the synthetic auxinic herbicide, the resistance was primarily governed by a single gene.^21^ For instance, in a dicamba‐resistant population of *Bassia scoparia* (L.) A. J. Scott, with the *KsIAA16R* resistance allele,^43^ the pattern of inheritance was consistent with a single gene model.^36^ Similar results have been reported for a 2,4‐D‐resistant *Sisymbrium orientale* L. with an in‐frame deletion mutation in the *IAA2* gene.^37^ Non‐target site mechanism of resistance to synthetic auxinic herbicides due to either enhanced herbicide metabolism or impaired herbicide translocation was primarily governed by a single gene in 2,4‐D‐resistant *Lactuca serriola* L.,^44^ *Raphanus raphanistrum* L.^32^ and *Sisymbrium orientale*.^45^ However, in *Galeopsis tetrahit* L., with two mechanisms of MCPA (4‐chloro‐2‐ethylphenoxyacetate) resistance, namely reduced herbicide translocation and enhanced rate of metabolism, the resistance trait was mediated by two nuclear genes with additive effects.^46^

The pattern of segregation can vary with the type of ploidy.^47^ In autotetraploid species, inheritance is complex as chromosomes either form multivalents or pair randomly during meiosis (tetrasomic inheritance).^48^ In contrast, in allotetraploid species, inheritance is similar to that of diploid species in which chromosomes form bivalents during meiosis (disomic inheritance).^49^ The segregation ratio of 3:1 observed in all F_2_ families can occur in both disomic and tetrasomic inheritance. However, due to the allopolyploid nature of *C*. album,^26^ we believe the 3:1 ratio represents the disomic inheritance for the studied populations in this research. In addition, this result suggests that the resistance allele is likely to present in one of the homoeologous chromosomes since the segregation pattern of the F_2_ families did not fit the 15:1 ratio, which represents the disomic inheritance when the resistance gene presents in both homoeologous chromosomes.^47^ This homoeologous heterozygosity has also been observed in other herbicide‐resistant polyploid weed species, *Avena fatua* L.^50^ and *Poa annua* L.^51^ In both cases, the resistance level conferred by the resistance gene was lower compared to that reported for unrelated diploid weed species (i.e. gene dosage effects). It is, however, unclear if homoeologous heterozygosity results in a lower resistance level to dicamba in *C. album*, as the mechanism of dicamba resistance in this species has yet to be completely elucidated. In our laboratory, work is currently in progress to understand the mechanism of dicamba resistance in *C. album*. Further research is needed to understand the impact of homoeologous heterozygosity on dicamba resistance in *C. album* once the mechanism of dicamba resistance is fully elucidated for this species.

## 4 CONCLUSION

The inheritance mode and segregation regularity in dicamba‐resistant *C. album* was studied in this research. The results showed that the resistance trait is dominant and can be transmitted by pollen. In addition, it appears that the resistance trait is governed by a single gene, and the segregation pattern follows the Mendelian laws for disomic inheritance, which is consistent with the allotetraploid origin of *C. album*. A single dominant gene mode of inheritance implies that the selection for dicamba resistance in *C. album* is more likely than if the resistance trait was polygenic. *Chenopodium album* is predominantly self‐pollinating. Hence, outcrossing by pollen could primarily contribute to the spread of the resistance gene in the local population rather than between populations. However, on a large scale, the spread of the resistance allele would primarily occur via seeds.

## Data Availability

The data that support the findings of this study are available from the corresponding author upon reasonable request.
